# Pediatric Migraine

**DOI:** 10.1155/2009/424192

**Published:** 2009-05-27

**Authors:** Ubaid Hameed Shah, Veena Kalra

**Affiliations:** Apollo Centre for Advanced Pediatrics, Indraprastha Apollo Hospital, New Delhi, India

## Abstract

Migraine is the most common cause of acute recurrent headaches in children. The pathophysiological concepts have evolved from a purely vascular etiology to a neuroinflammatory process. Clinical evaluation is the mainstay of diagnosis and should also include family history. Investigations help to rule out secondary causes. The role of new drugs in treatment of migraine is discussed and trials are quoted from literature. Indications for starting prophylaxis should be evaluated based on frequency of attacks and influence on quality of life. For management of acute attacks of migraine both acetaminophen and ibuprofen are recommended for use in children. Many drugs like antiepileptic drugs (AED), calcium channel blockers, and antidepressants have been used for prophylaxis of migraine in children. The data for use of newer drugs for migraine in children is limited, though AEDs are emerging a popular choice. Biofeedback and other nonmedicinal therapies are being used with promising results.

## 1. Introduction

Headache is a disabling neurological disorder of varied etiologies and a frequent health problem in children and adolescents [1]. Headaches in children can be a primary disorder or secondary to a number of causes. The four categories of primary headaches are: migraine, tension-type headache (TTH), cluster headache and trigeminal autonomic cephalalgias, and other primary headaches [2] (Table 1). Secondary headaches include conditions related to intracranial and extracranial infections, intracranial mass lesions, and head and neck trauma. Chronic recurrent headaches occur in approximately 40% of children at age of seven, increasing to 75% by the age of fifteen years. Pre-pubertal boys are more affected than girls, but after puberty, headaches are more common in girls [3]. Migraine is the most common cause and accounts for more than half the cases of recurrent headaches in children [4]. With such a high prevalence in school-aged children, migraine has significant effect on school attendance and family dynamics involved in caring for a sick child, with loss of job productivity and work attendance in parents [5].

## 2. Diagnostic Criteria of Pediatric Migraine

Pediatric migraine differs from adult migraine in presentation and response to treatment. It tends to be of shorter duration and is more often bilateral. Migraine is divided into six major categories (Table 1), the two most important of which are migraine without aura (Table 2) and migraine with aura. Under the new classification of 2004 [2], headaches of bilateral location and also those of shorter duration (one hour instead of two hours) are currently modified for migraine diagnosis in children. The criteria for ophthalmoplegic “migraine” now have been placed under “cranial neuralgias and central causes of facial pain,” and criteria for chronic migraine have been added. If a patient fulfils the criteria for more than one type of migraine, each type should be diagnosed.

## 3. Pathophysiology of Migraine

The pathophysiological concepts of migraine have advanced considerably over the last 20 years. The much popular vascular theory of migraine by Wolff has been undermined by phase model of migraine by Blau [6] and cerebral Doppler flow studies by Olesen et al. [7] who demonstrated that vasoconstriction did occur, but the timing of vasoconstriction did not precede the aura and continued well into the headache phase of the migraine. Migraine is now considered to originate in the brain, thus making it a neurological rather than vascular disease. According to this neurogenic theory the genetically sensitive migraine brain when exposed to a migraine-inducing environment undergoes neurochemical alterations resulting in premonitory symptoms. This alteration in neurochemical balance of the central nervous system leads to trigeminovascular activation with the release of vasoactive peptides and neurogenic inflammation. This in turn lowers the sensory threshold for trigeminal input entering the brainstem at the Nucleus Claudalis of the Trigeminal Nerve. Sensory input from the C1 and C2 dermatomes integrates with the trigeminal input, and eventually synapses in the somatosensory and limbic cortex, where it is interpreted into conscious awareness as headache. The characteristic form and development of sensory disturbances and demonstration of unique changes of brain blood flow during migraine auras suggest that the underlying mechanism is the spreading depression in cerebral cortex. The cortical spreading depression, which may be a key to an understanding of the migraine attack, is a short-lasting depolarization wave that moves across the cortex at a rate of 3–5 mm/min. A brief phase of excitation heralds the reaction which is immediately followed by prolonged nerve cell depression synchronously with a dramatic failure of brain ion homeostasis, efflux of excitatory amino acids from nerve cells, and enhanced energy metabolism [8]. 

Calcitonin gene-related peptide has been implicated in pathogenesis of migraine. Activation of trigeminal nerves releases CGRP and other peptides which release proinflammatory mediators. These mediators further increase CGRP synthesis and release over hours to days in correspondence with the 4- to 72-hour duration of a typical migraine episode. The increased CGRP synthesis and release might be mediated by activation of mitogen-activated protein kinase pathways, which, in turn, can be modulated by endogenous inflammatory substances such as TNF-alpha and affected by drugs such as sumatriptan.

It is now widely accepted that children with migraine have a genetic predisposition that is in some way activated by an environmental or physiological stimulus like exposure to drugs, diet, stress, puberty, and so forth. Major breakthrough was an identification of gene locus for familial hemiplegic migraine in the Cav2.1 subunit of the gene for the P/Q type, voltage gated calcium channel on chromosome 19. Since then many gene mutations have been identified in cases of familial hemiplegic migraine.

## 4. Evaluation of a Child with Headache

A detailed medical history is crucial. Assessment entails about characteristics of headache: location (unilateral or bilateral region); character (pulsating, pressing); severity and effect on ability to carry out daily activities; frequency and duration, including number of days missed from school; triggers; aggravating and relieving factors. Children with “acute headaches” should be questioned for possible trauma, symptoms suggestive of meningitis like fever, phono-photophobia, and stiffness of neck. In “chronic progressive headache” inquire about history of projectile vomiting, focal weakness, and systemic illness. History of coexisting medical disorders, emotional and social factors like academic failure, bullying, and so forth, symptoms of depression, family history of migraine, especially maternal, previous medications and their response, excessive use of multiple drugs, and history suggestive of drug-induced problems should always be part of medical history of a child with recurrent headaches.

A thorough general physical examination, blood pressure measurement, palpation of the head in search for sinus tenderness, nuchal rigidity, and visual examination should be done. Head circumference must be measured, even in older children, because progressive increases in intracranial pressure slowly cause macrocrania. Examine for neurocutaneous syndrome, particularly neurofibromatosis and tuberous sclerosis, which are highly associated with intracranial neoplasms. A detailed neurologic examination is essential to look for any objective evidence of organic causes of recurrent headaches. Fundus should be examined in chronic headache and suspected raised intracranial pressure.

## 5. Features Suggesting Migraine As Cause of Recurrent Headache in a Child

These include a positive family history, presence of trigger factors, relief by sleep, impairment of the child's social functioning, and presence of aura symptoms. Migraine in children is strongly associated with other childhood periodic syndromes, for example, cyclical vomiting, abdominal migraine, and benign paroxysmal vertigo. Very often, children with migraine frequently suffer from travel sickness and giddiness. Other strong associations are stress, depression, and psychiatric comorbidities. Migraine needs to be differentiated from Tension-type headache. Salient differentiating features are highlighted in Table 3.

## 6. Role of Investigations

In childhood recurrent headaches without neurological findings, current literature does not support performing routine laboratory studies, lumbar puncture, or EEG as part of the diagnostic evaluation. Investigate if history and examination points toward a secondary cause of headache. In 2002 American Academy of Neurology published practical guidelines for role of different investigations in children with recurrent headaches unassociated with trauma, fever, or other provocative causes [9].

## 7. Neuroimaging

Neuroimaging is not routinely necessary in recurrent headaches and a normal neurologic examination. Neuroimaging should be considered in children with an abnormal neurologic examination, coexistence of seizures, or both and if history suggests recent onset of severe headache, increasing frequency of headache, change in the type of headache, or subtle neurologic dysfunction (Table 4).

## 8. EEG

EEG is not recommended in the routine evaluation of a child with recurrent headaches, as it is unlikely to provide an etiology, improve diagnostic yield, or distinguish migraine from other types of headaches. Pooled data indicates that the EEG is either normal or demonstrates nonspecific abnormalities in most headache patients. Nonspecific abnormalities and benign epileptiform discharges are present in up to 10 percent of children with migraine, regardless of the diagnosis [10]. Current data does not suggest differences in EEG between children with migraine and nonmigraine type headaches which may be diagnostically helpful. Hemiplegic migraine has shown the most definite abnormal EEGs with a wide variety of patterns. During the ictus, severe unilateral or focal disturbances delta activity, theta-delta activity, theta activity or alpha-reduction are described. In most cases EEG changes subside in a few days and return to normal [11, 12].

## 9. Lumbar Puncture

Lumbar puncture should be done when a child with acute headache reveals signs of meningeal irritation or if there is high suspicion of meningitis on clinical grounds.

## 10. Treatment of Migraine

Treatment of pediatric migraine includes an individually tailored regimen for acute attack and prophylaxis of migraine using both nonpharmacologic and pharmacologic measures. The successful treatment involves explaining the disease process and reassuring the family.

## 11. Treatment of Acute Attacks of Migraine (Table 5)

### 11.1. Analgesics

Acetaminophen and Ibuprofen are safe, effective and widely used for treatment of acute attacks of migraine in children. The current evidence in literature shows that both are safe and effective in aborting the acute attack of migraine in children. Comparison of efficacy and safety at doses of 15 mg/kg acetaminophen and 10 mg/kg ibuprofen, respectively, found no significant differences [13]. Similarly there is no difference in efficacy, safety and tolerability between acetaminophen 15 mg/kg and Nimuselide 2.5 mg/kg [14]. Aspirin-containing compounds are of concern in children younger than 15 years because of the risk of Reye's syndrome. Although a combination of aspirin, caffeine, and acetaminophen is effective in adult acute migraine, it has not been tested in children for mild to moderate migraines.

### 11.2. Triptans

The triptans, selective serotonin 5-HT1B/1D agonists, are very effective acute migraine drugs. They are widely used in treatment of migraine attacks in adults and are very effective. However, children differ in respose to oral formulations of triptans as compared to adults. Oral treatment has been assessed with sumatriptan, rizatriptan, and zolmitriptan and found to be without benefit [15–18]. In one trial of 32 patients zolmitriptan was superior to placebo [19]. There is inadequate data for effectiveness of subcutaneous sumatriptan in children. In adolescents, only intranasal administration has demonstrated efficacy, for both sumatriptan and zolmitriptan [20–22].

## 12. Other Medications for Acute Migraine Attacks

Other class of drugs used widely for treatment of migraine attacks is ergot groups but current evidence finds no difference in effect between oral dihydroergotamine and placebo [23]. Prochlorperazine is more effective than ketorolac in the reduction of symptoms 1 hour after intake in controlling severe acute migrane attack in emergency department [24].

## 13. Prevention of Migraine Attacks

The indications for use of migraine prophylaxis in children include missing more than 3 days of school for a month or having 1 to 2 headaches for a week that interfere with performing daily activities. Unresponsiveness to symptomatic treatment, failure of non-pharmacological measures to improve headache frequency, and/or presence of basilar or hemiplegic migraine are also appropriate indications for preventive therapy [25]. Many drugs have been used for prevention of migrainous attacks in children, but there is paucity of evidence to support their use in general. Commonly used drugs for prevention of migraine attacks are tabulated in Table 6.

## 14. Beta Blockers and Calcium Channel Blockers

Beta-blockers are one of the most commonly prescribed drugs for the prevention of migraine. Some controlled trials have shown good results in adult [26]. Initial pediatric trials showed inconsistent results with mixed success. Recent Cochrane data base review found propranolol to be effective for prophylaxis of pediatric migraine [27]. The side effects of propranolol including insomnia, weight gain, tiredness, and depressive symptoms often limit their role as prophylactic agents in children. 

Among the calcium channel blockers only flunarizine has shown consistent efficacy and safety as prophylactic agent for paediatric migraine [27]. American academy of neurology's recommendations concluded that Flunarizine is probably effective for prophylaxis though it is not available in the United States.

## 15. Anticonvulsant Therapy

There is growing interest in the use of anticonvulsant drugs in prophylaxis of migraine. In children, small group studies have shown efficacy. Caruso et al. [28] reported that 31 children aged 7 to 16 years were responsive to Valproic acid in the 15–45-mg/kg dosage range, with 76% of patients having a greater than 50% reduction in headache frequency, while 18% had a greater than 75% reduction, and 6% were headache-free. A study using standardized doses of either 500 mg or 1000 mg of sodium divalproate in 9- to 17-year-old children also reported reduction in severity on the 10 point Visual Analog Scale from 6.8 to 0.7, with a decrease in headache frequency from 6 per month to 0.7 per month [29]. Winner et al. [30] reported effectiveness of topiramate in children and adolescents in dose of 2-3 mg/kg/day (maximum dose 200 mg) with reduced mean monthly migraine frequency from 5.4 days per month to 1.9 days per month. One retrospective study assessed the efficacy and safety of levetiracetam for pediatric migraine at doses of 125–250 mg twice daily and found that the mean frequency of headache attacks fell from 6.3 to 1.7/month and 52% of patients experienced elimination of migraine attacks during treatment. No side effects were reported in 82.4% but 10.5% discontinued treatment because of side effects including somnolence, dizziness, and irritability.

Cochrane review concluded that anticonvulsants appear to be both effective in reducing migraine frequency and reasonably well tolerated. There was noticeable variation among individual agents, but there are insufficient data to know whether this is due to chance or variation in true efficacy. Other anticonvulsant drugs like acetazolamide, clonazepam, lamotrigine, and vigabatrin do not produce results superior to placebo [31].

## 16. Other Medications for Prophylaxis of Migraine

Cyproheptadine, an antihistamine with serotonine blocking properties, has been used for migraine prophylaxis in children in doses of 2 to 4 mg/day showing reduction in headache parameters with only few side effects like weight gain and sedation. Amitriptyline, at a dose of 1 mg/kg per day, has also shown effectiveness in open-label trials. But further trials are needed to recommend these drugs for routine use in children [32].

## 17. Nonpharmacological or Behavioral Therapy

The importance of nonmedicinal treatment deserves review. The treatment methods include categories like promoting adherence, education of the patient as well as maintaining healthy lifestyle habits. These healthy lifestyle habits include maintenance of adequate fluid hydration, regular exercise programs, not skipping meals, eating a balanced healthy diet, and maintaining adequate sleep. Abstract reports have demonstrated that skipping meals and sleep alterations contribute to frequent headaches in adults and children, and maintenance of healthy lifestyle habits may help overall improve the outcome of childhood headache disorders. Biobehavioral guidelines are under development, and further study of the effectivenss of biobehavioral management is needed [33].

## 18. Biofeedback

Biofeedback is a technique intended to teach patients self-regulation of certain physiologic processes not normally considered to be under voluntary control. The technique involves the feedback of a variety of types of information not normally available to the patient, followed by a concerted effort on the part of the patient to use this feedback to help alter the physiological process in some specific way. The type of feedback used in an intervention depends on the nature of the disease or disorder under treatment. For migraine headaches, EMG measuring contraction of the frontalis muscle and skin temperature feedback data are used (thermal biofeedback). Thermal biofeedback is an effective technique used by many migraine patients to reduce the pain intensity and frequency of their headaches. This is especially true of pediatric migraineurs, particularly those who have entered puberty. Patients achieve control through a combination of visualization, voluntary relaxation, and mechanical feedback. In this technique, a temperature sensor is placed on the finger, and the subject is taught to increase the temperature, an effect that is mediated through peripheral vasodilation. Biofeedback training is done either in individual or group sessions, alone or in combination with other behavioral therapies designed to teach relaxation. A typical program consists of 10 to 20 training sessions of 30 minutes each. Training sessions are performed in a quiet, nonarousing environment. Subjects are instructed to use mental techniques to affect the physiologic variable monitored. Typically, some type of reward system is incorporated for successful alteration of the feedback parameter. This reward may be in the form of sensory signals such as lights or tone, verbal praise, or other pleasant stimuli. At-home thermal biofeedback practice is frequently more successful in children because they tend to be more imaginative than adults. Relaxation training and biofeedback have proven to be promising treatments for children with migraine headaches. Feedback training was accompanied by significant reduction of cortical excitability. This was probably responsible for the clinical efficacy of the training; a significant reduction of days with migraine and other headache parameters was observed. It is suggested that normalization of the threshold regulation of cortical excitability during feedback training may result in clinical improvement [34].

## Figures and Tables

**Table 1:** International Classification of Headache Disorders.

Migraine
Migraine without aura
Migraine with aura
Childhood periodic syndromes that are commonly precursors of migraine
Retinal migraine
Complications of migraine
Probable migraine

Tension-type headache (TTH)

Infrequent episodic tension-type headache
Frequent episodic tension-type headache
Chronic tension-type headache
Probable tension-type headache

Cluster headache and other trigeminal autonomic cephalalgias

Cluster headache
Paroxysmal hemicrania
Short-lasting unilateral neuralgiform headache attacks with conjunctival injection and tearing (SUNCT)
Probable trigeminal autonomic cephalalgia

Other primary headaches

Primary stabbing headache
Primary cough headache
Primary exertional headache
Primary headache associated with sexual activity
Hypnic headache
Primary thunderclap headache
Hemicrania continua
New daily-persistent headache (NDPH)

**Table 2:** Diagnostic criteria for Pediatric migraine without aura.

A	≥5 attacks fulfilling features B to D
B	Headache attack lasting 1 to 72 hours

C	Headache has at least 2 of the following 4 features:
	(1) Bilateral or unilateral (frontal/temporal) location
	(2) Pulsating quality
	(3) Moderate to severe intensity
	(4) Aggravated by routine physical activity

D	At least one of the following accompanies headache:
	(1) Nausea and/or vomiting
	(2) Photophobia and phonophobia (may be inferred from their behavior)

**Table 3:** Differentiating Migraine from Tension-type headache.

Characteristics	Migraine	Tension-type headache
Pain features of acute attacks	Throbbing	Boring or squashing
	Mostly unilateral	Usually bilateral
	Worsening of pain with head movement	No effect of head movement

Associated features	Nausea or vomiting	None
	Photophobia and phonophobia	

Triggering factors	Altered sleep patterns (too little or too much)	Psychological stress
	Skipping meals	
	Overexertion	
	Change in stress level (too much or relaxation)	
	Excess afferent stimuli (such as bright lights)	
	Menstruation	

**Table 4:** Indications for neuroimaging in children with headache.

Acute headache
Chronic-progressive pattern
Focal neurologic symptoms
Abnormal neurologic examination
Presence of neurocutaneous syndrome
Changing pattern of headache
Age younger than three years

**Table 5:** Treatment of acute attacks of migraine.

Class	Drug	Comments
Analgesics	Ibuprofen, PO 7.5–10 mg/kg	First line drug; safe and effective in children
	Acetaminofen, PO 15 mg/kg	Comparable efficacy and safety profile with ibuprofen
	Nimuselide, PO 2.5 mg/kg	

Triptans	Sumatriptan Nasal spray, 5 mg, 20 mg	Easy administration, faster initial relief, and more side effects as compared to placebo
	Subcutaneous, 0.06 mg/kg	Administration difficulty, chest and neck discomfort, and reported side effects
	Oral, 50 to 100 mg	Not effective
	Rizatriptan, PO, 5 mg	Studied in adolescent; adverse effects reported were fatigue, dizziness, somnolence, dry mouth, and nausea
	Zolmitriptan, PO 2.5–5 mg	Evaluated in 12–17 years

Other medication	Ketorolac IV 0.5 mg/kg; maximum 30 mg	IV prochlorperazine is superior to IV ketorolac in the acute treatment of migraine headaches in emergency department
	prochlorperazine IV (0.15 mg/kg; maximum 10 mg)	

**Table 6:** Drugs used for prevention of migraine attacks.

Class	Drug & dosage	Comments
Beta blockers	Propranolol 3 mg/kg/day	Reduced energy, tiredness, postural symptoms, contraindicated in asthma, depressive side effects, often limits their usefulness in children

Calcium chanel blockers	Flunarizine, PO, 5 mg	Not available in US
	Nimodipine	Not recommended for use in pediatric migraine

Anticonvulsants	Valproate 15–45 mg/kg/day, PO	Drowsiness, weight gain, tremor, hair loss, fetal abnormalities,haemato-logical or liver abnormalities
	Topiramate 2–3 mg/kg/day, PO	Found to be effective in pediatric migraine. Side effects include cognitive changes, weight loss, and sensory symptoms
	Levetiracetam 250–500 mg	Evaluated in pediatric migraine and found to be effective. About 10% children report somnolence, dizziness, and irritability

Antidepressents	Amitriptyline 1 mg/kg/day, PO	Reduces headache frequency and severity; sedation major side effect
	Trazodone 1 mg/kg/day, divided TID, PO	Current literature: no evidence of benifit
	Pizotefen	Current literature: no evidence of benifit
